# Clathrin‐mediated EGFR endocytosis as a potential therapeutic strategy for overcoming primary resistance of EGFR TKI in wild‐type EGFR non‐small cell lung cancer

**DOI:** 10.1002/cam4.3635

**Published:** 2020-12-12

**Authors:** Boyeon Kim, Young Soo Park, Jae Sook Sung, Jong Won Lee, Saet Byeol Lee, Yeul Hong Kim

**Affiliations:** ^1^ Cancer Research Institute Korea University College of Medicine Seoul Republic of Korea; ^2^ BK21 Plus program Korea University College of Medicine Seoul Republic of Korea; ^3^ Department of Oncology/Hematology Korea University Anam Hospital Seoul Republic of Korea

**Keywords:** clathrin mediated endocytosis, gefitinib, non‐small cell lung cancer, resistance

## Abstract

**Objectives:**

Oncogenic alterations of epidermal growth factor receptor (EGFR) signaling are frequently noted in non‐small cell lung cancer (NSCLC). In recent decades, EGFR tyrosine kinase inhibitors (TKIs) have been developed, although the therapeutic efficacy of these inhibitor is restricted to EGFR‐mutant patients.

In this study, we investigated that clathrin‐mediated EGFR endocytosis hampers the effects of gefitinib and sustains NSCLC cells with wild‐type EGFR.

**Materials and Methods:**

NSCLC cell lines (H358, Calu‐3, SNU‐1327, and H1703) were stimulated with the EGF and treated with gefitinib and endocytosis inhibitors (phenylarsine oxide (PAO) and Filipin III). Growth inhibition and apoptosis were evaluated. Immunofluorescence, immunoprecipitation, and western blot assay were performed to investigate EGFR endocytosis and determine the signaling pathway. Xenograft mouse models were used to verify the combination effect of gefitinib and PAO in vivo.

**Results:**

We confirmed the differences in EGFR endocytosis according to gefitinib response in wild‐type EGFR NSCLC cell lines. EGFR in gefitinib‐sensitive and ‐refractory cell lines tended to internalize through distinct routes, caveolin‐mediated endocytosis (CVE), and clathrin‐mediated endocytosis (CME). Interestingly, while suppressing CME and CVE did not affect cell survival in sensitive cell lines significantly, CME inhibition combined with gefitinib treatment decreased cell survival and induced apoptosis in gefitinib‐refractory cell lines. In addition, blocking CME in the refractory cell lines led to downregulate of p‐STAT3 and inhibit nuclear localization of STAT3 in vivo, combination treatment with gefitinib and a CME inhibitor resulted in tumor regression accompanying apoptosis in xenograft mouse models.

**Conclusion:**

Clathrin‐mediated EGFR endocytosis contribute primary resistance of gefitinib treatment and CME inhibition combined with gefitinib could be an option in treatment of wild‐type EGFR NSCLC.

## Graphical abstract

We found that refractoriness with EGFR TKI treatment in wild‐type EGFR NSCLC cell lines was attributable to a survival mechanism through clathrin‐mediated EGFR endocytosis. Blocking clathrin‐mediated EGFR endocytosis could be a therapeutic strategy for overcoming resistance to gefitinib treatment in wild‐type EGFR NSCLC.

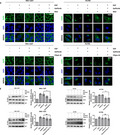

## 1 INTRODUCTION

Lung cancer is a leading cause of cancer‐related deaths worldwide and non‐small cell lung cancer (NSCLC) represents more than 80% of all lung cancer cases.^1^, ^2^ The treatment of NSCLC generally includes chemotherapy, targeted therapy, immunotherapy, or a combination of these therapies.^3^, ^4^

In the last decade, researchers have uncovered the molecular determinants of lung cancer and identified numerous nonoverlapping driver genomic events including epidermal growth factor receptor (EGFR), KRAS, ALK, ROS1, and HER2.^5^ EGFR is a transmembrane receptor tyrosine kinase protein and expressed in some normal epithelial, mesenchymal, and neurogenic tissue. After its ligand binding, EGFR transduces information from the microenvironment into the cell through downstream signaling pathways.^6^, ^7^ However, aberrant EGFR activation is frequently reported in diverse human malignancies, including NSCLC, and dysregulation of EGFR is associated with worse clinical outcomes such as a reduced survival rate, frequent metastasis to other organ, and poor chemosensitivity.^8^, ^9^, ^10^

Recently, a paradigm in cancer therapies is treating oncogene positive cells through targeting a single‐oncogene alteration. Two types of EGFR‐targeting drugs have been developed, monoclonal antibodies (mAbs) and tyrosine kinase inhibitors (TKIs),^11^ and development of EGFR TKIs in particular was an important milestone in the targeted therapy of NSCLC.^3^, ^12^ The mAbs, such as cetuximab, have been approved as a treatment for progressive colorectal cancer patients, as well as head and neck cancer patients.^13^, ^14^ However, cetuximab currently has no role in treatment of NSCLC patients, because of its marginal clinical benefit.^15^ Gefitinib is the first EGFR TKI approved for clinical use in lung cancer patients. EGFR TKIs have been recommended as a potential first‐line treatment for advanced NSCLC patients with somatic EGFR TK domain mutations such as exon 19 deletions (E746‐A750 (2235‐2249del), E746‐A750 (2236‐2250del), L747‐E749 A750P (2239–2247 and 2248del), etc.), and a exon 21 point mutation (L858R).^3^, ^16^, ^17^ However, the frequency of these mutations has been reported as only 10%–40% in lung cancer patients depending on various factors including smoking history, sex, and ethnicity.^18^, ^19^ Therefore, most NSCLC patients who have wild‐type EGFR do not receive the benefits from this treatment but are instead given highly toxic general anticancer drugs.^20^, ^21^ Nevertheless, 10%–20% of lung cancer patients with wild‐type EGFR gained a therapeutic advantage from EGFR TKIs treatment,^18^, ^22^, ^23^ which implied that there are underlying molecular mechanisms that determine the efficacy of EGFR TKIs and are independent of EGFR somatic mutations.

Canonical EGFR signaling is initiated by the binding of a ligand, such as EGF or TGF‐α, followed by phosphorylation‐mediated TK activation of intracellular signaling proteins. The PI3K‐AKT and RAS‐RAF‐MEK‐ERK pathways are the main EGFR signaling axis for cancer cell proliferation, chronic initiation, progression, and angiogenesis.^24^, ^25^ In our previous study, we observed that phosphorylation of AKT and ERK was inhibited by gefitinib in both gefitinib‐sensitive and ‐insensitive wild‐type EGFR NSCLC cell lines. These results suggest that an unknown mechanism outside of the main axis of EGFR signal transduction is exist and contributes to the response to gefitinib, and we proposed EGFR endocytosis as a potential mechanism that affects to cell survival and sensitivity to gefitinib.^26^

In the current study, we determined that clathrin‐mediated EGFR endocytosis is involved in gefitinib refractoriness in wild‐type EGFR NSCLC cell lines. Moreover, we confirmed that this primary resistance can be overcame by combination therapy with a CME inhibitor and gefitinib using in vitro and in vivo models.

## 2 MATERIALS AND METHODS

### 2.1 Reagents and antibodies

Gefitinib (AstraZeneca), Phenylarsine Oxide (PAO) (Sigma‐Aldrich), Filipin III (Sigma‐Aldrich), anti‐EGFR (sc‐373746), anti‐EGFR (4267, Cell signaling), anti‐p‐EGFR (2234, Cell signaling), anti‐STAT3 (sc‐8019), anti‐p‐STAT3 (sc‐8059), anti‐ERK (9102, Cell signaling), anti‐p‐ERK (9101, Cell signaling), anti‐EEA1 (610457, BD Biosciences), anti‐PARP (9542, Cell signaling), anti‐c‐Myc (9402, Cell signaling), anti‐β‐actin (A5316), Goat anti‐mouse IgG (H + L)‐HRP conjugate (1706516, Bio‐Rad), Goat anti‐rabbit IgG polyclonal HRP conjugated (ADI‐SAB‐300, Enzo), Alexa Fluor 488 goat anti‐rabbit antibody (A32731, Thermo Fisher Scientific), and Alexa Fluor 594 goat anti‐mouse antibody (A32742, Thermo Fisher Scientific).

### 2.2 NSCLC cell lines and cell culture

The H358, Calu‐3, and H1703 were purchased from the American Type Culture Collection (ATCC). The SNU‐1327 were purchased from the Korean Cell Line Bank (Seoul, South Korea). The H358, SNU‐1327, and H1703 cell lines were cultured in Roswell Park Memorial Institute (RPMI) −1640 medium (GE Healthcare) with 10% of fetal bovine serum (FBS) (GE Healthcare) and 1% of penicillin‐streptomycin solution (GE Healthcare). The Calu‐3 cell line was cultured in Eagle's minimum essential medium (EMEM) with 10% of FBS, 1% of penicillin‐streptomycin solution, and 1% of GlutaMAX (Thermo Fisher Scientific) All cell lines were incubated in a humidified incubator at 37°C with 5% of CO_2_.

### 2.3 Cell proliferation assay

After drug treatment, cells were incubated in 96‐well plate with complete medium and CCK‐8 solution (Dojindo laboratories) according to the manufacturer's guide. The absorbance was detected at 450 nm using an iMark microplate reader (Bio‐Rad). The experiments are repeated three time independently. The half maximal inhibitory concentration (IC50) were determined using SoftMax Pro software (Molecular Devices Corp.). Combination effect on cell survival was analyzed through the Combination Index (CI) using CompuSyn software (Biosoft). CI defines synergism (CI<1), additive effect (CI = 1), and antagonism (CI > 1).^27^

### 2.4 Caspase‐3/7 activity

Apoptotic cell death was measured with an Apo‐ONE Homogeneous Caspase‐3/7 assay kit (Promega) according to the manufacturer's guide. The absorbance was detected at 520 nm using a SpectraMax i3x microplate reader (Molecular Devices). The experiments are repeated three time independently.

### 2.5 Western blotting

Cells were harvested, washed with 1x phosphate‐buffered saline (PBS), and lysed with Radioimmunoprecipitation assay buffer (RIPA) buffer containing PhosSTOP and a protease inhibitor cocktail (Sigma‐Aldrich). The total protein concentration was determined using the Bradford assay (Bio‐Rad) with bovine serum albumin (BSA) (Sigma‐Aldrich) as the standard. The protein samples were electrophoresed on an 8%–12% of sodium dodecyl sulfate‐polyacrylamide gel and transferred to a polyvinylidene difluoride (PVDF) membrane (GE Healthcare) and blocked with 5% of nonfat milk/1x Tris‐buffered saline, 0.1% of Tween 20. The membranes were incubated with primary antibodies for overnight at 4℃ and were washed and incubated with the secondary antibody for 1 hour at room temperature. The protein bands were visualized using ECL (pico/femto) reagents (Thermo Fisher Scientific) and detected using a ChemiDoc imaging system (Bio‐Rad). The experiment was independently repeated three times and the intensity of protein bands was calculated by with Image J software.

### 2.6 Immunoprecipitation

To isolate EGFR in early endosomes, immunoprecipitation was conducted as previously described.^28^ For immunoprecipitation, 1 mg of protein lysate was incubated with Protein A/G agarose beads (Thermo Fisher Scientific) and anti‐EEA1 antibody for 10 h. The beads were then washed thoroughly three times with lysis buffer, and the proteins were extracted in sample buffer by boiling for 10 min. Western blot assays were then performed as described above.

### 2.7 Nuclear fractionation

To separate the nuclear fraction, we conducted subcellular fractionation. The protocol is based on that from Dr. Richard Patten at Abcam. Concisely, cells were washed with 1xPBS, and lysed with subcellular fractionation buffer. Lysate were passed through a 25 Ga needle syringe 10 times and leave on ice for 20 min. Centrifuge at 3000 rpm for 5 min and get the nuclear pellets. After adding SF buffer, the nuclear pellets pass through a 25 Ga needle again. Centrifuge again at 3000 rpm for 10 min and remove the supernatant. Resuspend the nuclear pellets in the nuclear lysis buffer (RIPA buffer with 10% glycerol and 0.1% SDS).

### 2.8 Immunofluorescence

NSCLC cells were cultured on cover slides in complete medium. Before analysis, the cells were starved for 24 hours and pretreated for 30 min with each inhibitor (Gefitinib, PAO, or Filipin III). The cells were fixed with 4% of paraformaldehyde (PFA) for 10 min and permeabilized with 0.25% of Triton X‐100 in PBS for 30 min. The cells were incubated with 5% of BSA for 1 hour. The primary antibodies for EGFR, EEA1, and STAT3 were diluted 1:200 with a 5% of BSA solution and incubated with the samples at 4°C overnight. A 1:1000 dilution of Alexa Fluor 488 goat anti‐rabbit secondary antibody and Alexa Fluor 594 goat anti‐mouse were incubated with the samples for 1 h in the dark. The cell nucleus was stained with 1 μg/ml of 4,6‐diamidino‐2‐henylindole (DAPI) (Sigma‐Aldrich) for 10 min. The cells were washed three time with 1× PBS‐T between each step. The samples were observed using an LSM800 confocal microscope (Zeiss).

### 2.9 Xenograft mouse model

Male BALB/c nu/nu mice (20 g, 6 weeks old) were purchased from Orient Bio (Gyeonggi‐do). 5 × 10^6^ SNU‐1327 cells with Matrigel (BD Biosciences) were subcutaneously injected into the mice. The mice were randomly divided into four groups. After 100 mm^3^ tumor sizes, the mice were treated with the vehicle control (0.5% Tween 80 in PBS for oral treatment, 0.1% DMSO in PBS for intraperitoneal treatment), oral gefitinib (5 mg/kg, daily), intraperitoneal PAO (0.2 mg/kg, every 2 days), or the combined drugs for 3 weeks and checked the tumor volume twice per week. Tumor volumes were calculated by the following formula: volume (mm^3^) = width^2^ × length/2. All procedures were performed according to the guidelines of the Institutional Animal Care and Use and IRB committees (KUIACUC‐2015‐13) at Korea University (Seoul, Korea).

### 2.10 Immunohistochemistry

Immunostaining for Ki‐67 and terminal deoxynucleotidyl transferase dUTP nick end labeling assay (Millipore) were performed using the Polink‐2 Plus HRP Broad DAB detection system (GBI Labs) according to the manufacturer's instructions. Images of the stained cells were obtained using an Olympus BX51 microscope (Olympus) and DP Manager software (Olympus).

### 2.11 Statistical analysis

The results are expressed as the mean ± standard deviation (SD). Significant differences between values under different experimental conditions were determined using the paired or unpaired Student's *t* test, and *p* < 0.05 were considered statistically significant.

## 3 RESULTS

### 3.1 Gefitinib‐sensitive and ‐refractory wild‐type EGFR NSCLC cell lines differ in EGFR endocytosis

**FIGURE 1:**
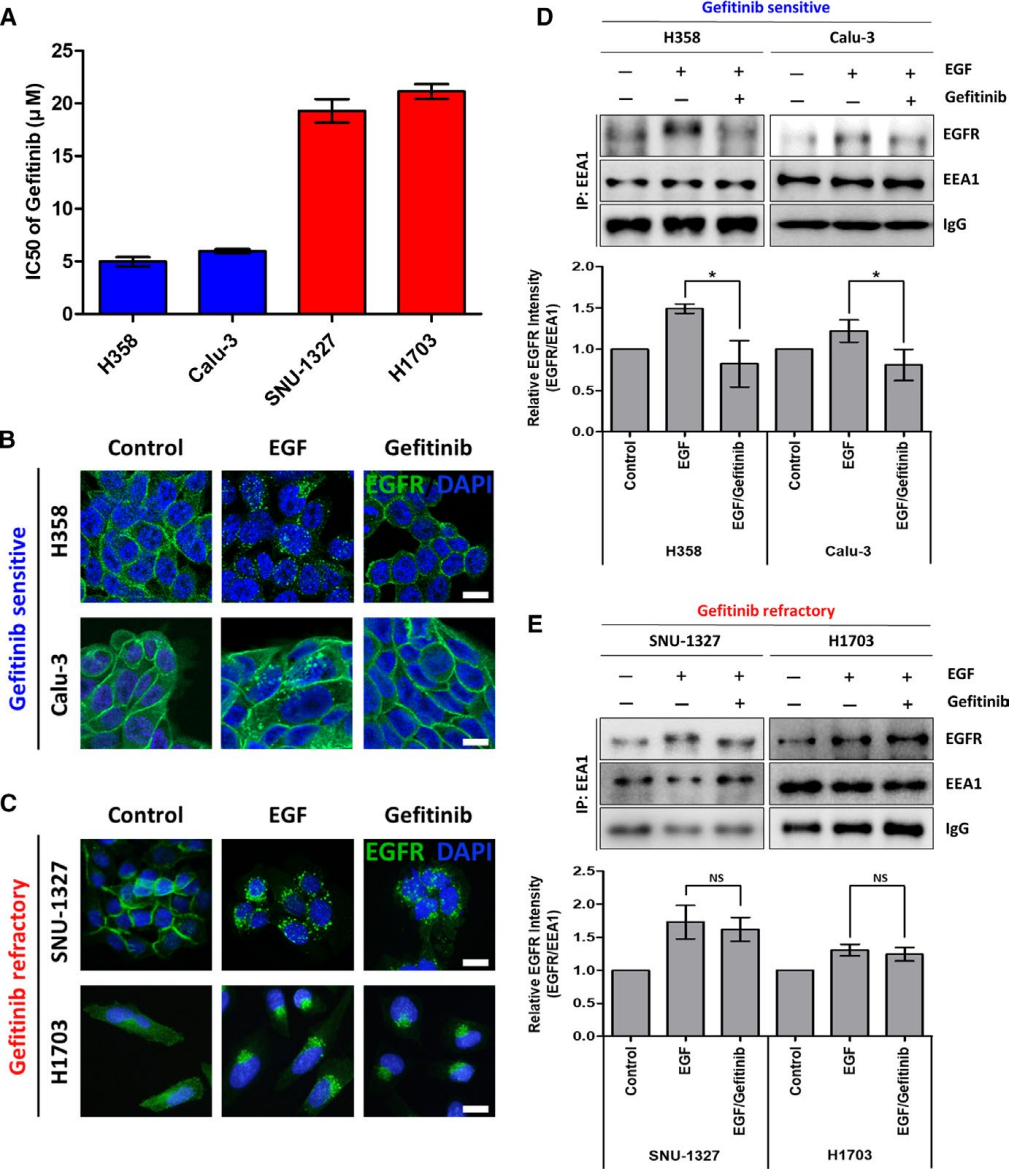
Effects of gefitinib on EGFR endocytosis in non‐small cell lung cancer with wild‐type EGFR. (A) The IC50 values of gefitinib in four NSCLC cell lines (H358, Calu‐3, SNU‐1327, and H1703) were determined using the CCK‐8 assay. The cells were treated with gefitinib for 48 h. (B, C) Confocal microscopy images of the gefitinib‐sensitive H358 and Calu‐3 cell lines (B) and gefitinib‐refractory SNU‐1327 and H1703 cell lines (C); EGFR (green) and DAPI (blue). The cells were starved for 24 h in serum‐free medium, pretreated with 5 μm gefitinib for 30 min and treated with 100 ng/ml EGF for 10 min. Scale bar: 20 μm (D, E) Immunoprecipitation with anti‐EEA1 antibody was performed in the gefitinib‐sensitive cell lines H358 and Calu‐3 (D) and gefitinib‐refractory cell lines SNU‐1327 and H1703 (E). The immunoblots were detected with EGFR and EEA1 antibodies (top panels), and the EGFR intensity relative to EEA1 (EGFR/EEA1) was calculated (bottom panels). Each bar represents the mean value of three experiments with SD. NS, not significant, *p < 0.05

To confirm whether EGFR endocytosis is associated with gefitinib sensitivity in wild‐type EGFR NSCLC cell lines, we used four cell lines; H358, Calu‐3, SNU‐1327, and H1703. We first classified these cell lines into gefitinib‐sensitive and ‐refractory groups according to their IC50 concentration for gefitinib, which was determined using growth tests with several concentration of gefitinib. The H358 and Calu‐3 cell lines exhibited decreased viability at relatively lower concentrations of gefitinib (IC50 mean value 4.98 μm, 5.96 μm, respectively, sensitive group)^29^, ^30^ compared to SNU‐1327 and H1703 (IC50 mean value 19.3 μm, 21.13 μm, respectively, refractory group) (Figure 1A).

We next compared the internalization of EGFR with or without EGF or gefitinib treatment between the two groups. As shown as Figure 1B,C EGFR basally localized at the cytoplasmic membrane in all cell lines. With EGF treatment, punctate green spots near the nucleus and faded EGFR staining in the membrane were detected, indicating EGFR internalization through vesicles. Interestingly, gefitinib treatment resulted in clear differences in EGFR endocytosis between the sensitive group and the refractory group. In the sensitive group, EGFR accumulated on the cytoplasmic membrane after gefitinib treatment (Figure 1B). However, in the refractory group, EGFR was still internalized to the cytosol following gefitinib treatment (Figure 1C).

We also isolated early stage endosomes via immunoprecipitation using an anti‐EEA1 (early endosome marker) antibody and quantified the EGFR protein bands using western blotting. Consistently, the amount of EGFR in the early endosomes was increased by EGF treatment in both groups and abated by gefitinib treatment only in the sensitive group (Figure 1D). In contrast, the amount of EGFR in the early endosomes was maintained following gefitinib treatment in the refractory group (Figure 1E).

### 3.2 EGFR endocytosis in gefitinib‐refractory cell lines is dependent on a clathrin‐mediated pathway

**FIGURE 2:**
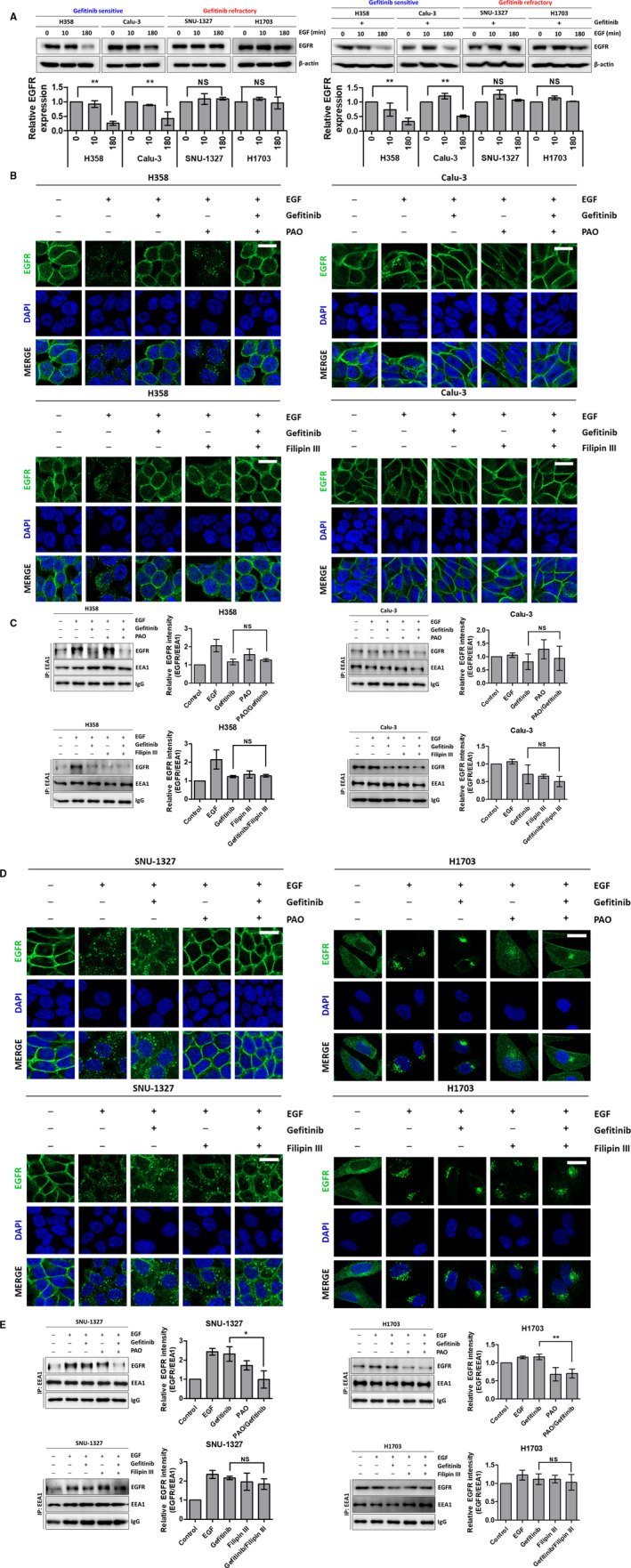
Blocking clathrin‐mediated endocytosis inhibits EGFR endocytosis in gefitinib‐refractory cell lines. (A) Gefitinib‐sensitive (H358 and Calu‐3) and ‐refractory (SNU‐1327 and H1703) cell lines were starved for 24 h, pretreated without (left panel) or with (right panel) gefitinib (5 μm) for 30 min, then, treated with EGF (100 ng/ml) for 0, 10, or 180 min. The cells were harvested to western blot for EGFR and β‐actin. (B, C) Confocal microscopy images (B) and immunoprecipitation (C) of the gefitinib‐sensitive cell lines H358 (left panels) and Calu‐3 (right panels). Each cell line was starved for 24 h, pretreated without or with gefitinib (5 μm), PAO (0.1 μm), or Filipin III (1 μg/ml) for 30 min, then, treated with EGF (100 ng/ml) for 10 min. (D, E) Confocal microscopy images (D) and immunoprecipitation (E) of the gefitinib‐refractory cell lines SNU‐1327 (left panels) and H1703 (right panels). The microscopy images were stained for EGFR (green) and DAPI (blue), and the immunoblots were detected with anti‐EGFR and anti‐EEA1 antibodies. Scale bar: 20 μm

Because we observed distinct EGFR localization between the two groups, we confirmed that there was a difference in the fate of EGFR (recycling or degradation) after EGF binding in the gefitinib‐sensitive and ‐refractory groups. Following EGF treatment for 10 min, the total amount of EGFR was not notably different among the four cell lines. However, after 180 min of EGF treatment, EGFR in the sensitive group was degraded, but EGFR in the refractory group remained intact (Figure 2A, left). In addition, a comparable result was observed with gefitinib treatment (Figure 2A, right). This finding indicates that EGFR fate resulting from the different EGFR localization in both groups is associated with mechanisms other than the canonical EGFR tyrosine kinase activity.

CME and CVE of EGFR are important mechanisms that determine the fate of EGFR in terms of recycling or degradation.^31^, ^32^ To monitor EGFR internalization in the sensitive and refractory groups under CME‐ or CVE‐dependent conditions, we used the CME inhibitor PAO and the CVE inhibitor Filipin III at a concentration that efficiently blocked EGFR endocytosis. As shown in Figure 2B, PAO treatment in sensitive group resulted in EGFR internalization. However, this spatial change in EGFR was suppressed following Filipin III treatment. Also, gefitinib combined with each inhibitor suppressed EGFR uptake in a manner similar to gefitinib treatment alone (Figure 2B; Figure S1A). This result is consistent with immunoprecipitation data of the amount of EGFR in early endosomes (Figure 2C; Figure S1B). However, in contrast with the sensitive group, PAO treatment and PAO‐gefitinib combination treatment reduced EGFR endocytosis and the amount of EGFR in endosomal vesicles to the baseline in the refractory group (Figure 2D,E; Figure S1A,B).

### 3.3 Blocking clathrin‐mediated endocytosis reduces the viability of gefitinib‐refractory cell lines

**FIGURE 3:**
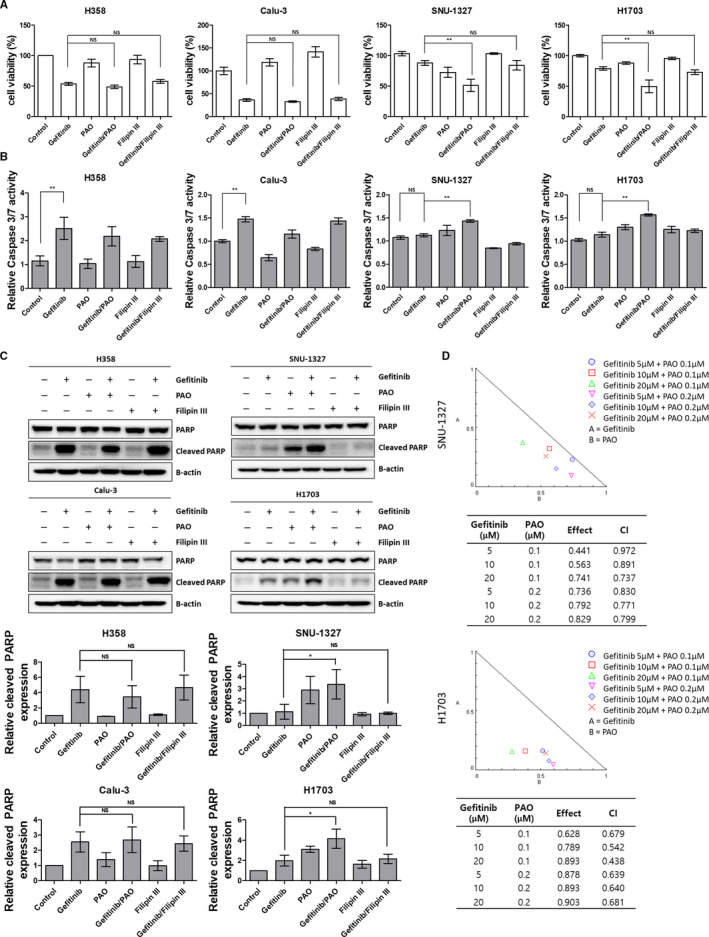
CME inhibition promotes apoptosis in gefitinib‐refractory cell lines. (A, B) Viability assays (CCK‐8) (A) and the Apo‐ONE assay (B) were performed in H358, Calu‐3, SNU‐1327, and H1703 cell lines after treatment with gefitinib (5 μm), PAO (0.1 μm), and Filipin III (1 μg/ml) for 48 h. Each bar represents the mean value of three independent experiments with SD. NS, not significant, **p < 0.01. (C) The change in the expression level of cleaved PARP was evaluated using western blotting. Cells were treated with gefitinib (5 μm), PAO (0.1 μm), and Filipin III (1 μg/ml) for 48 h. (D) Combination Index value was marked at isobologram. CCK‐8 assay was performed in SNU‐1327 and H1703 cell lines after treatment with gefitinib (5, 10 and 20 μm) and PAO (0.1 and 0.2 μm) for 48 h

Considering that CME is an essential mechanism for sustained EGFR signaling and the results presented above, we postulated that blocking CME would increase the gefitinib sensitivity of the refractory cell lines. To determine whether the inhibition of clathrin‐dependent EGFR endocytosis could overcome primary resistance in wild‐type EGFR NSCLC cell lines, cell survival rates were determined using the CCK‐8 assay. Gefitinib markedly reduced cell proliferation in the sensitive group; however, endocytosis inhibitors did not significantly affect cell survival. Contrarily, cell survival slightly diminished in the refractory group after gefitinib treatment. Also, as expected, CME inhibition and gefitinib combination treatment reduced cell survival but CVE inhibition did not affect cell survival (Figure 3A). In addition, an Apo‐ONE assay to evaluate caspase‐3/7 activity showed a robust induction of apoptosis with gefitinib treatment in the sensitive group but not in the refractory group. In the refractory group, apoptosis was induced by PAO treatment and PAO‐gefitinib combination treatment (Figure 3B). This observation was supported by the increased levels of cleaved PARP, an apoptotic marker (Figure 3C). Also, we confirmed combination effect in diverse concentration of gefitinib (5, 10, and 20 μm) and PAO (0.1 and 0.2 μm) in gefitinib‐refractory cell lines. As shown as Figure 3D, combination treatment with gefitinib and PAO showed synergy effect (CI < 1).

### 3.4 Inhibition of CME reduces nuclear STAT3 localization and c‐Myc expression in gefitinib‐refractory cell lines

**FIGURE 4:**
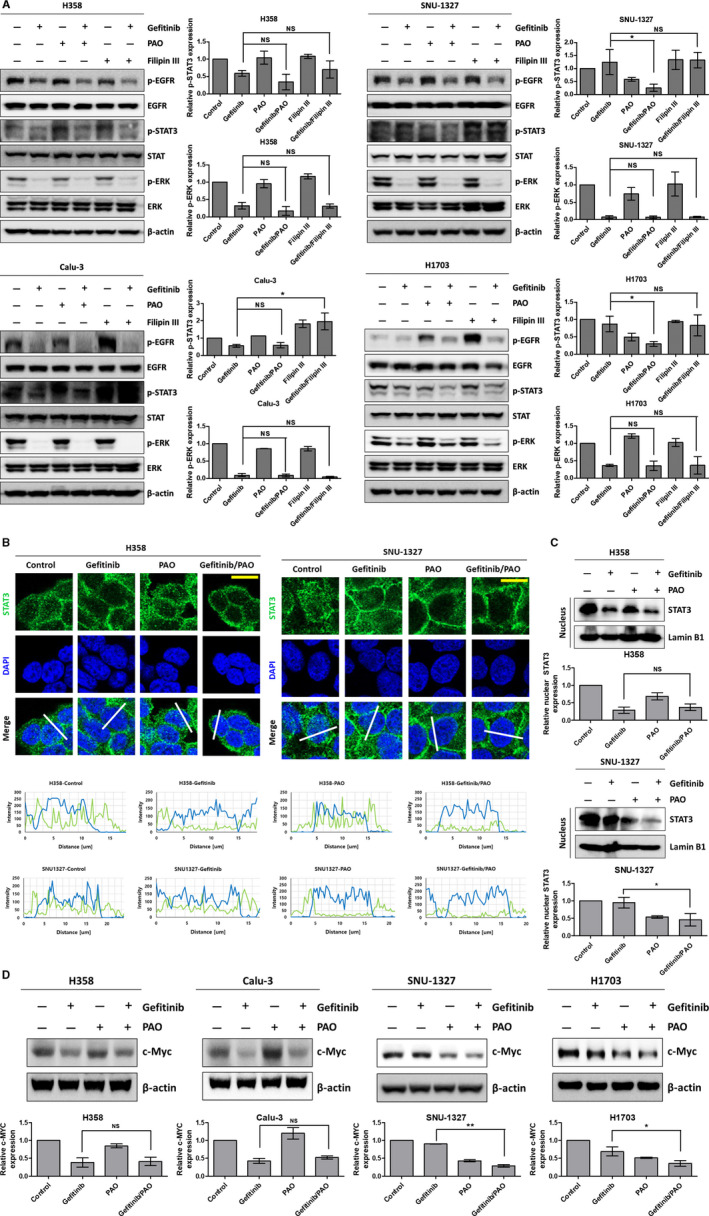
CME inhibition induces degradation of p‐STAT3 and hampers nuclear localization of STAT3. (A) The effects of endocytosis inhibitors on phosphorylation of EGFR (Y1068), STAT3 (Y705), and ERK (T202/Y204) were evaluated using western blotting. Cells were treated with gefitinib (5 μm), PAO (0.1 μm), and Filipin III (1 μg/ml) for 30 min. (B) Nuclear localization of STAT3 was investigated in the H358 and SNU‐1327 cell lines using immunofluorescence staining. Cells were treated with gefitinib (5 μm) and PAO (0.1 μm) for 30 min then stained for STAT3 (green) and DAPI (blue). Immunofluorescence images (top panels) were obtained using confocal microscopy, and intensity values was analyzed using ZEN software along with a white line in each image (bottom panels). Scare bar (yellow line): 20 μm. (C) Nuclear STAT3 was investigated in the H358 and SNU‐1327 cell lines by western blot. (D) The expression of c‐Myc, a target gene of STAT3, was determined using western blotting. Cells were treated with gefitinib (5 μm) and PAO (0.1 μm) for 24 h

To determine the signaling pathway influencing cell survival in gefitinib‐refractory cell lines, we confirmed phosphorylation of STAT3, a downstream signaling protein of EGFR. Although tyrosine phosphorylation of STAT3 was blocked by gefitinib in the sensitive group, the p‐STAT3 level was maintained with the same concentration of gefitinib in the refractory group. However, at that concentration of gefitinib, p‐ERK was downregulated in all cell lines. Interestingly, PAO or PAO‐gefitinib combination treatment, but not Filipin III, reduced p‐STAT3 levels in the refractory cell lines (Figure 4A). The results shown in Figures 3 and 4A indicate that phosphorylation of STAT3 is involved in the survival of refractory cell lines following gefitinib treatment, and downregulation of STAT3 can lead to apoptosis due to CME inhibition.

When we investigated regulation of nuclear STAT3, we observed that translocation for nuclear localization of STAT3 was blocked by gefitinib in the sensitive group but not in the refractory group. However, PAO and PAO‐gefitinib combination treatment suppressed the localization of STAT3 into the nucleus in the refractory group (Figure 4B,C). In addition, c‐Myc, a transcriptional target of STAT3, expression was decreased by PAO or PAO‐gefitinib combination treatment in the refractory cell lines (Figure 4D).

### 3.5 Inhibition of CME reverses the gefitinib response in vivo

**FIGURE 5:**
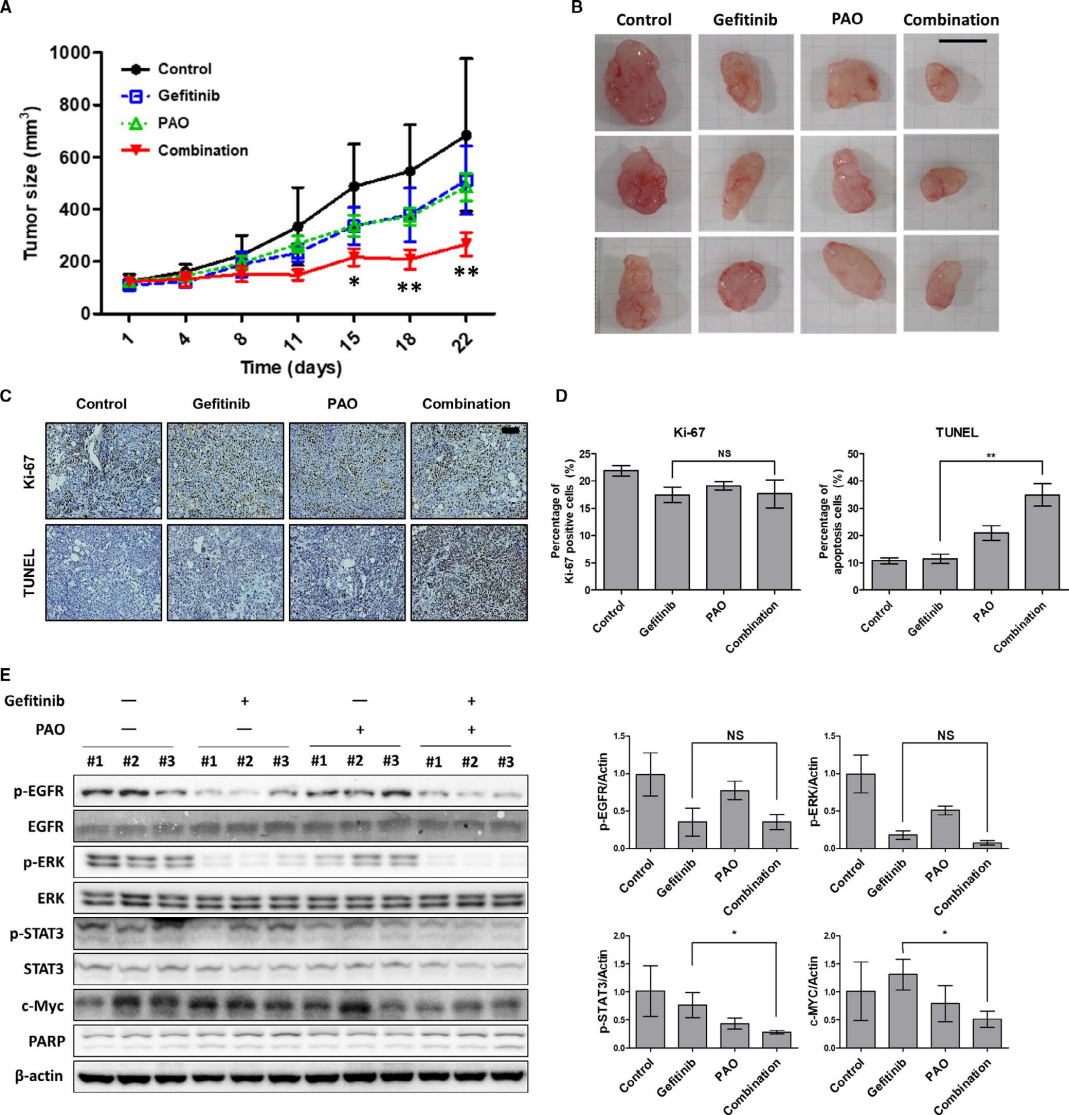
Combination treatment with CME inhibitors and gefitinib impairs tumor growth in vivo. (A) The gefitinib‐refractory SNU‐1327 cell line was subcutaneously injected in BALB/c‐nude mice. The mice were treated with 0.5% of Tween 80 in PBS (control), oral gefitinib (5 mg/kg daily), intraperitoneal PAO (0.2 mg/kg every 2 days), or a combination. Tumor volumes were measured for 3 weeks. Mean value of tumor volume were plotted with SD. *p < 0.05. (B) Images of resected tumors. Three representative tumors in each group were presented. Scale bar: 1 cm. (C) Representative tumor tissue sections in each mouse group is stained for Ki‐67 and TUNEL staining. Scale bar: 200 μm. (D) The intensity of Ki‐67‐ and TUNEL‐positive cells was analysis using Image J software. NS, not significant, **p < 0.01. (E) Phosphorylation of EGFR (Y1068), STAT3 (Y705), and ERK (T202/Y204), as well as c‐Myc and PARP expression, were evaluated with western blotting using the lysate of three tumors from each mouse group

Based on our in vitro findings, SNU‐1327 cells (5x10^6^) were subcutaneously injected in the flank of BALB/c‐nude mice. These mice were divided into four groups of control, gefitinib treatment, PAO treatment, and combination treatment, and monitored for 3 weeks. The tumor growth rate was decreased in the PAO/gefitinib combination treatment group compared with the PAO or gefitinib single‐treatment groups (Figure 5A,B). In the histological analysis, the antiproliferative effects and apoptosis induced by combination treatment with PAO and gefitinib were comparable to the gefitinib group (Figure 5C,D). Also, combination treatment significantly reduced the expression of phospho‐STAT3 and c‐Myc compared with gefitinib treatment (Figure 5E).

## 4 DISCUSSION

A number of recent reports have shown that proper endocytic uptake and endosomal sorting of signaling receptors are crucial mechanisms for the regulation of cellular behaviors including growth, development, and differentiation.^33^, ^34^, ^35^ Also, abnormal expression and dysregulation of intracellular trafficking machinery can lead to the development of cancers.^35^, ^36^

After EGFR is activated upon EGF binding, EGFR can be internalized through diverse endocytic carriers and transported to early endosomes. EGFR endocytosis have been thought to a cellular mechanism that terminates activated signaling or recycles back to the cell surface for continued signaling.^37^, ^38^ The internalization of EGFR consequently affects cell growth and survival via interactions with various adaptors or signaling molecules. Also, EGFR can localize to organelles, such as nucleus and mitochondria, and trigger the transcription of diverse proteins for cell survival.^33^, ^39^, ^40^ However, the therapeutic relevance of EGFR endocytosis in NSCLC has not been disclosed thoroughly.

In this study, we found for the first time that low sensitivity to gefitinib in wild‐type EGFR NSCLC cell lines was attributable to a survival mechanism through clathrin‐mediated EGFR endocytosis.

Interestingly, we found that the EGFR was degraded in gefitinib‐sensitive cells, whereas EGFR in refractory cells was recycled and maintained at a steady amount regardless of gefitinib treatment. Because different endocytic mechanisms are involved in EGFR sorting, namely CME and CVE^32^, we investigated the role of these mechanisms in gefitinib reactivity. EGFR endocytosis can be distinguished according to the concentration of EGF. At low EGF concentrations, most EGFR are internalized through CME, but EGFR internalization occurs through both CME and CVE at high EGF concentrations.^32^, ^41^ To involve both pathways, we used a high EGF concentration and showed that differences in the EGFR endocytosis mechanism of gefitinib‐refractory and ‐sensitive cells. Also, we showed that CME inhibition contributed to cell death and increased gefitinib sensitivity in wild‐type EGFR gefitinib‐refractory cells.

A recent study reported that degradation of mutant EGFR (exon 19 deletion, L858R, T790M, and C797S) and impairment of its signaling are caused by CME inhibition. In addition, the authors suggested that reactivation of mutant EGFR degradation through clathrin inhibition could overcome the resistance to EGFR TKIs.^42^ In short, CME is an important mechanism in prolonging the duration of EGFR signaling not only in wild‐type EGFR NSCLC cell lines, but also in mutant EGFR NSCLC cell lines.

To investigate the signaling pathways that sustain gefitinib‐refractory cell lines independent of AKT and ERK, we examined the STAT3 pathway. The JAK/STAT cascade pathway is a side branch of the EGFR signaling pathway and activates cell signaling for survival, differentiation, and cell migration. Phosphorylation of STAT3 persisted in refractory cells following gefitinib treatment, in contrast with sensitive cells, but the CME inhibitor and gefitinib combination hindered STAT3 phosphorylation in refractory cells. Also, protein expression of c‐Myc, a target gene of STAT3,^43^, ^44^ was reduced following PAO and gefitinib combination treatment. STAT3 is a downstream signaling pathway of p38, and according to recent research, p38‐mediated phosphorylation of the C‐terminus of EGFR induces CME of the unliganded EGFR monomers and EGFR is recycled back to the cytoplasmic membrane.^45^ The data from this study, taken together with previous studies, provides a meaningful clue regarding the role of STAT3 in CME and the reactivity of EGFR TKIs, although further study is necessary.

We confirmed meaningful insight that CME could contribute to treating lung cancer, but some limitations remain for EGFR endocytosis studies. Currently, there is no specific targeting drug for EGFR endocytosis that approved for clinical use. Therefore, further research regarding EGFR endocytosis and the development of endocytosis inhibitors for wild‐type EGFR NSCLC patients are needed.

Consequently, we provided a novel insight that blocking clathrin‐mediated EGFR endocytosis could be a therapeutic strategy for overcoming resistance to gefitinib in wild‐type EGFR NSCLC and new evidence of EGFR signaling mechanism via CME.

## CONFLICT OF INTEREST

The authors declare no competing financial interests.

## AUTHOR CONTRIBUTIONS

Study concept and design, YHK, BK, YSP, JSS; conducting the experiments, BK; analysis and interpretation of data, YHK, BK, YSP, JSS, JWL SBL; drafting of the manuscript, BK; study supervision, YHK. All authors read and approved the final version.

## Supporting information

Fig S1
