# Supplementary material for: Clathrin‐mediated EGFR endocytosis as a potential therapeutic strategy for overcoming primary resistance of EGFR TKI in wild‐type EGFR non‐small cell lung cancer
Source: Cancer Med. 2020 Dec 12;10(1):372–85. doi: 10.1002/cam4.3635 (PMC7826488; doi:10.1002/cam4.3635)
Supplement: Supplementary file 1 — Fig S1 [file CAM4-10-372-s001.docx]

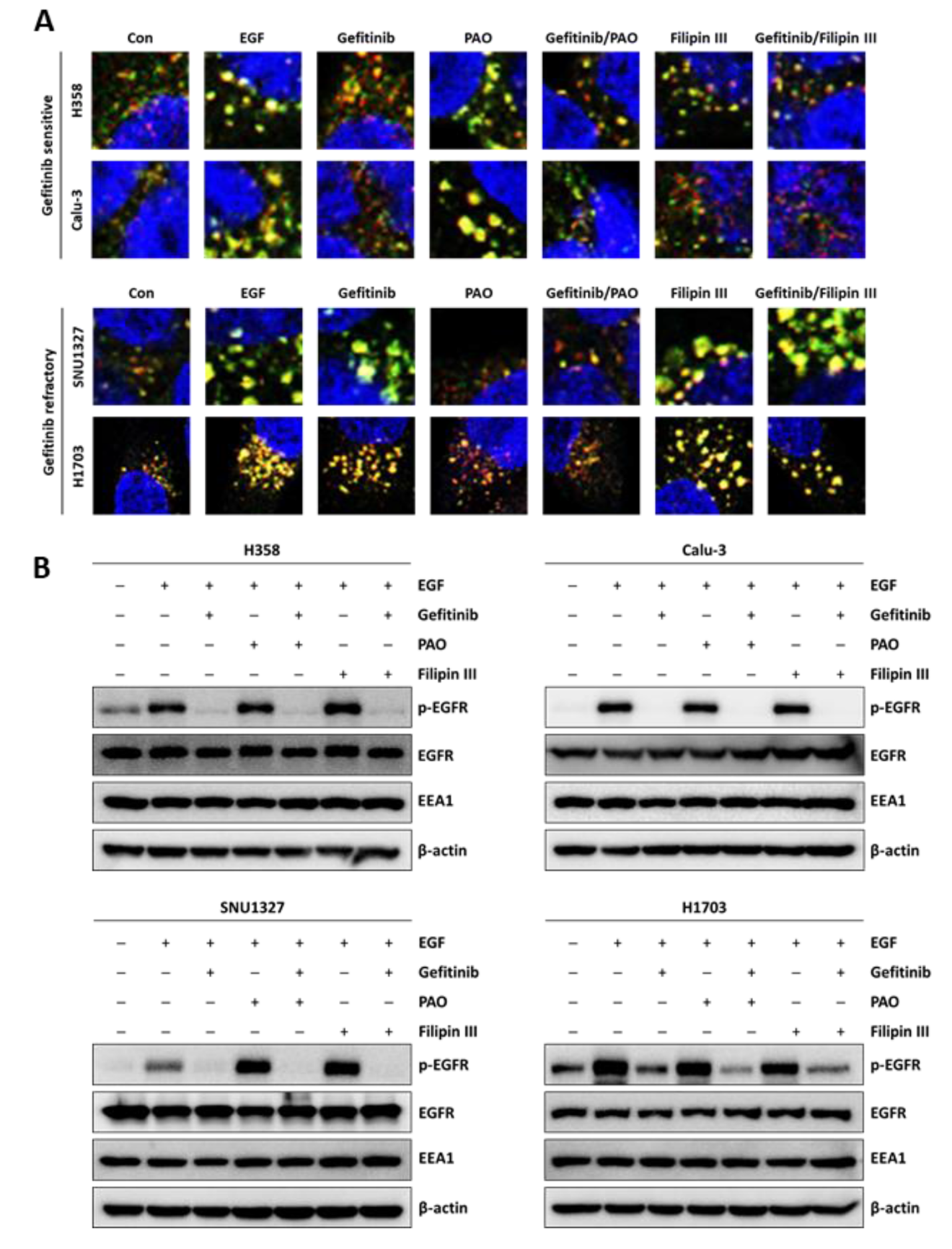


**Supplementary figure S1.** Total amount of EGFR and EEA1 and co-localization. (A) Gefitinib-sensitive (H358 and Calu-3) and -refractory (SNU-1327 and H1703) cell lines were starved for 24 hours, pretreated without or with gefitinib (5 μM), PAO (0.1μM), or Filipin III (1 μg /mL) for 30 min, then treated with EGF (100 ng/mL) for 10 min. The confocal microscopy images were stained for EGFR (green), EEA1 (red) and DAPI (blue). (B) The cells were then subjected to western blotting for p-EGFR (Y1068), EGFR, EEA1 and β-actin.
